# S1P Released by SGPL1-Deficient Astrocytes Enhances Astrocytic ATP Production via S1PR_2,4_, Thus Keeping Autophagy in Check: Potential Consequences for Brain Health

**DOI:** 10.3390/ijms24054581

**Published:** 2023-02-26

**Authors:** Shah Alam, Sumaiya Yasmeen Afsar, Gerhild Van Echten-Deckert

**Affiliations:** LIMES Institute for Membrane Biology and Lipid Biochemistry, Kekulé-Institute, University of Bonn, 53115 Bonn, Germany

**Keywords:** sphingosine 1-phosphate (S1P), S1P-lyase (SGPL1), SPLIS, glucose metabolism, autophagy

## Abstract

Astrocytes are critical players in brain health and disease. Sphingosine-1-phosphate (S1P), a bioactive signaling lipid, is involved in several vital processes, including cellular proliferation, survival, and migration. It was shown to be crucial for brain development. Its absence is embryonically lethal, affecting, inter alia, the anterior neural tube closure. However, an excess of S1P due to mutations in S1P-lyase (SGPL1), the enzyme responsible for its constitutive removal, is also harmful. Of note, the gene *SGPL1* maps to a region prone to mutations in several human cancers and also in S1P-lyase insufficiency syndrome (SPLIS) characterized by several symptoms, including peripheral and central neurological defects. Here, we investigated the impact of S1P on astrocytes in a mouse model with the neural−targeted ablation of SGPL1. We found that SGPL1 deficiency, and hence the accumulation of its substrate, S1P, causes the elevated expression of glycolytic enzymes and preferentially directs pyruvate into the tricarboxylic acid (TCA) cycle through its receptors (S1PR_2,4_). In addition, the activity of TCA regulatory enzymes was increased, and consequently, so was the cellular ATP content. The high energy load activates the mammalian target of rapamycin (mTOR), thus keeping astrocytic autophagy in check. Possible consequences for the viability of neurons are discussed.

## 1. Introduction

The brain contains the highest lipid content in the body, apart from the adipose tissue [1], and the highest sphingolipid content [2]. Although lipids constitute 50% of its dry weight, the mammalian brain depends upon glucose as its main source of energy [3]. Glucose metabolism provides the fuel for ATP generation but also delivers intermediates for the biosynthesis of lipids, nucleic acids, and amino acids [4]. In the brain, glucose metabolism and the mammalian target of rapamycin (mTOR) signaling pathway are intimately linked [5]. The latter regulates several cellular processes, including autophagy, a cellular “recycling” pathway that involves the lysosomal degradation of cytoplasmic proteins or entire organelles for the catabolic regeneration of nutrient pools [6]. Considerable evidence indicates that autophagy plays critical roles in glia and neurons that affect the development, functionality, and viability of the nervous system [7,8]. Among several factors known to affect brain autophagy, sphingolipid metabolism plays an ambiguous regulatory role [9].

Sphingosine-1-phosphate (S1P), an evolutionarily conserved catabolic intermediate of sphingolipid metabolism, exerts multiple cellular functions, either as a ligand of a subfamily of five G-protein-coupled receptors (S1PR_1–5_) or acting intracellularly as a second messenger [10]. Compelling experimental evidence indicates the crucial role of S1P in the regulation of diverse fundamental processes in the brain, including neural development, differentiation, migration, and survival [11,12]. The content of S1P in the brain exceeds by far the amount of this lipid in the liver or spleen [13]. S1P concentrations in different brain regions are regulated by the level of its synthesis from sphingosine, mainly by sphingosine kinase 2 (SK2) [14], and by its degradation via S1P phosphatases [12] and S1P-lyase (SGPL1), which irreversibly cleaves S1P to ethanolamine phosphate (EAP) and hexadecenal [15]. In humans, SGPL1 is encoded by *SGPL1*, which has been shown to harbor autosomal recessive mutations causing a variety of pathologies, including peripheral and central neurological defects, collectively referred to as S1P-lyase insufficiency syndrome (SPLIS) [16].

While there is no doubt regarding the essential role of S1P in brain development [17,18], its role in neurodegenerative processes is still ambiguous and divisive [11,19]. Several studies reveal a neuroprotective function of S1P in the brain [20,21,22], while other reports instead argue in favor of a neurotoxic effect of S1P [23,24,25]. In an attempt to clarify the function of S1P in the brain, we have generated a mouse model in which SGPL1 is inactivated specifically in neural cells (SGPL1^fl/fl/Nes^), thus causing a marked accumulation of its substrate, S1P, in the brain [26]. As a result, neurons’ synaptic architecture and plasticity were significantly affected [26]. Moreover, neuronal autophagy was blocked in its early stages, leading to the accumulation of neurodegenerative biomarkers, along with behavioral changes, illustrated by deficits in cognitive skills as well as in motor coordination, in SGPL1^fl/fl/Nes^ mice [26,27]. Furthermore, S1P accumulation in neurons caused elevated levels of cytosolic calcium and the hyperphosphorylation of tau at disease-relevant sites [28]. In response to neuronal damage, microglial cells were activated, showing impaired autophagy and propagating neuroinflammation [29]. At the molecular level, astrocyte-derived S1P was found to play a key role in the activation of microglia in neural-targeted SGPL1-deficient brains [29]. In this follow-up study, we investigated the effect of SGPL1 deficiency and hence S1P accumulation in astrocytes. Given the essential role of astrocytic glucose metabolism [30] and autophagy in neuronal health [31], and based on previous results indicating the critical effect of S1P metabolism on glucose breakdown in fibroblasts [32] and on neural autophagy [27,33,34], we focused on these two processes in primary cultured astrocytes lacking SGPL1. We found that S1P triggers glucose catabolism via S1PR_2,4_ and channels pyruvate into the tricarboxylic acid (TCA) cycle at the expense of lactate formation. The increased generation of ATP favored mTOR activation, thus negatively affecting astrocytic autophagy.

## 2. Results

### 2.1. Neural SGPL1 Ablation Triggers Glucose Metabolism via S1P Receptors 2 and 4 in Primary Cultured Astrocytes

As glucose is the primary energy source for the brain, and astrocytes are mostly glycolytic [35], we first investigated the expression of two key glycolytic enzymes, (i) phosphofructokinase (PFK), the rate-limiting enzyme of glycolysis, and (ii) glyceraldehyde-3-phosphate dehydrogenase (GAPDH), which, due to its constant expression in most cell types, is often used as a housekeeping reference.

**Figure 1:**
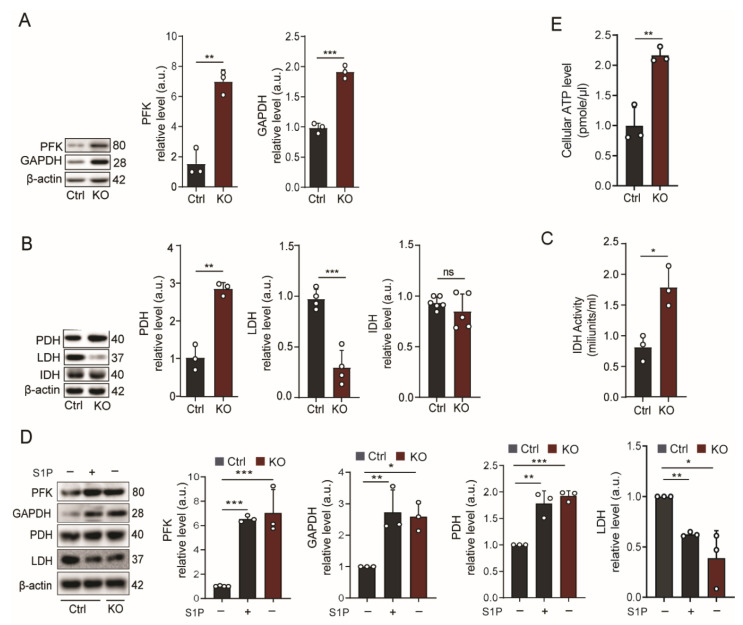
Neural SGPL1 ablation triggers glucose degradation in primary cultured astrocytes. (**A**,**B**) Protein quantification of phosphofructokinase (PFK), glyceraldehyde-3-phosphate dehydrogenase (GAPDH), pyruvate dehydrogenase (PDH), lactate dehydrogenase (LDH), and isocitrate dehydrogenase (IDH) in astrocytes from control (Ctrl) or SGPL1^fl/fl/Nes^ (KO) mice, as indicated. (**C**) IDH activity measurement. (**D**) Protein quantification of the indicated enzymes following stimulation (+) of control astrocytes with exogenous S1P (10 nM, 24 h), (−) represents without stimulation (**E**) Quantification of ATP in cultured astrocytes derived from control (Ctrl) and SGPL1^fl/fl/Nes^ (KO) mice. For all, representative immunoblots are shown with β-actin as loading control. Bars represent means ± SEM, n ≥ 3, unpaired Student’s *t*-test, one-way ANOVA with Bonferroni multiple comparison test; * *p* < 0.05, ** *p* < 0.001, *** *p* < 0.0001, ns, not significant.

As shown in Figure 1A, the protein levels of both PFK and GAPDH were considerably increased in primary cultured SGPL1-deficient astrocytes, suggesting a substantial elevation in glycolysis. We then evaluated the two enzymes that are decisive in the anaerobic (lactate dehydrogenase (LDH)) or aerobic (pyruvate dehydrogenase (PDH)) fate of pyruvate. As shown in Figure 1B, the substantial reduction in LDH expression of approximately 70% is paralleled by a more than 2-fold increase in the expression of PDH in SGPL1-deficient astrocytes. This result indicates that pyruvate is mainly oxidatively decarboxylated to acetyl-CoA at the expense of its reduction to lactate. As acetyl-CoA is further metabolized via the TCA cycle, we determined the expression and activity of isocitrate dehydrogenase (IDH), one of the points of regulation of the TCA cycle. While the expression of IDH was not changed (Figure 1B), its activity significantly increased by nearly 100% (Figure 1C). Notably, the results obtained in astrocytes lacking SGPL1 could be recapitulated by incubating control astrocytes for 24 h in the presence of 10 nM S1P (Figure 1D). This result confirms our previous finding that the amount of S1P secreted into the extracellular medium by SGPL1-deficient astrocytes is significantly higher than that found in the control medium, amounting to about 0.7 pmol/mg of cell protein [29]. We then determined the amount of ATP in control and SGPL1-deficient astrocytes. As ATP production via the TCA cycle is more efficient (30 ATP per glucose molecule) than glycolysis (2 ATP/glucose), we expected an elevation in the ATP content in SGPL1-deficient astrocytes. As shown in Figure 1E, this was indeed the case, as astrocytes derived from SGPL1^fl/fl/Nes^ mice exhibited a significantly higher amount (more than twofold) of ATP (Figure 1E).

**Figure 2:**
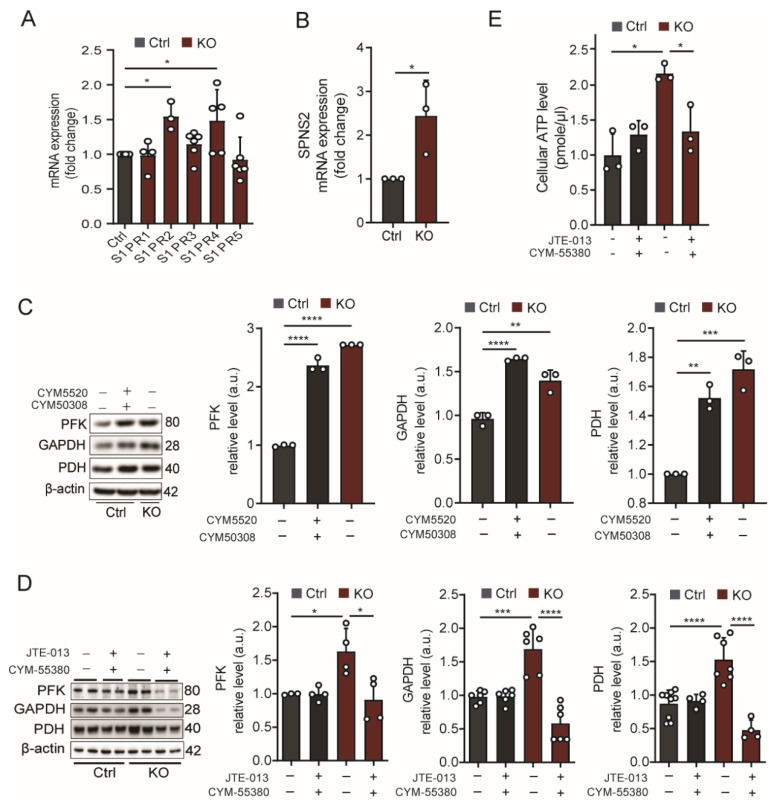
S1P receptors 2 and 4 mediate the effect of S1P on glucose degradation and ATP generation in SGPL1-deficient astrocytes. (**A,B**) Transcript quantification of the five S1P receptors (S1PR) and of the S1P transporter SPNS2 in SGPL1-deficient astrocytes relative to control cells (Ctrl) by qPCR using β-actin as a reference. (**C**) Protein quantification of PFK, GAPDH, and PDH following stimulation (+) of control astrocytes with a combination of specific agonists of S1PR_2_ (5 µM CYM5520) and S1PR_4_ (5 µM CYM50308) for 24 h. (**D**) Protein quantification of PFK, GAPDH, and PDH in astrocytes from control (Ctrl) or SGPL1^fl/fl/Nes^ (KO) mice cultured for 24 h in the absence (−) or presence (+) of the S1PR_2_ antagonist JTE-013 (10 µM) and the S1PR_4_ antagonist CYM-55380 (10 µM) as indicated. (**E**) Determination of ATP levels in control (Ctrl) or SGPL1-deficient astrocytes (KO) in the absence or presence of S1PR_2,4_ antagonists as indicated. For all, representative immunoblots are shown with β-actin as loading control. Bars represent means ± SEM, n ≥ 3, unpaired Student’s *t*-test, one-way ANOVA with Bonferroni multiple comparison test; * *p* < 0.05, ** *p* < 0.001, *** *p* < 0.0001, **** *p* < 0.00001.

**Figure 3:**
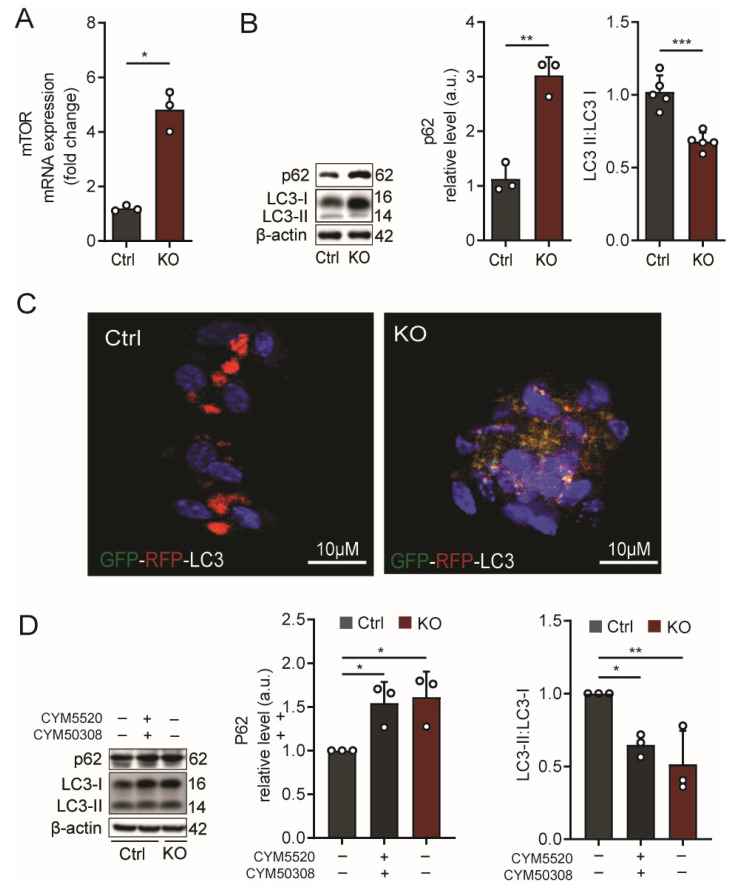
S1P activates mTOR and inhibits autophagy via S1PR_2,4_ in SGPL1-deficient astrocytes. (**A**) Transcript quantification of mTOR in SGPL1-deficient astrocytes (KO) relative to controls (Ctrl) by qPCR using β-actin as reference. (**B**) Quantification of autophagy marker proteins p62 and LC3-II:LC3-I and (**C**) representative images showing the fluorescence of the RFP–GFP–LC3 construct in cultured astrocytes from control (Ctrl) and SGPL1^fl/fl/Nes^ (KO) mice. Red puncta represent autolysosomes, whereas yellow puncta represent autophagosomal structures. DAPI staining indicates cell nuclei in blue. Scale bar: 50 µm. (**D**) Protein quantification of p62, LC3-I, and LC3-II following stimulation (+) of control astrocytes with a combination of specific agonists of S1PR_2_ (5 µM CYM5520) and S1PR_4_ (5 µM CYM50308), (−) represents without stimulation for 24 h. For all, representative immunoblots are shown with β-actin as loading control. Bars represent means ± SEM, n ≥ 3, unpaired Student’s *t*-test; one-way ANOVA with Bonferroni multiple comparison test, * *p* < 0.05, ** *p* < 0.001, *** *p* < 0.0001.

**Figure 4:**
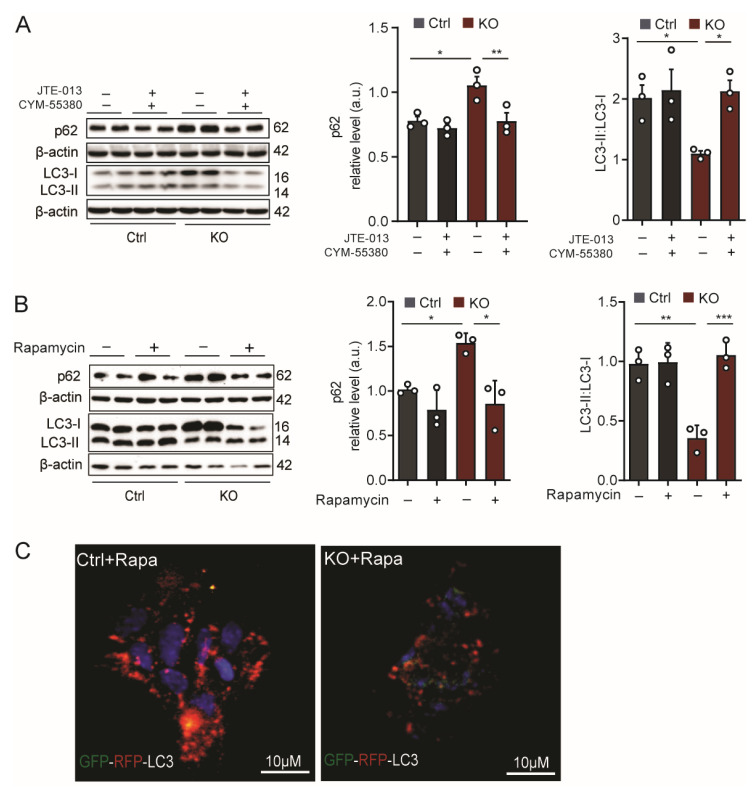
Rapamycin and S1PR antagonists re-establish autophagy in SGPL1-deficient astrocytes. (**A**) Quantification of autophagy marker proteins p62 and LC3-II:LC3-I in astrocytes from control (Ctrl) or SGPL1^fl/fl/Nes^ (KO) mice cultured for 6 h in the absence (−) or presence (+) of the S1PR_2_ antagonist JTE-013 (10µM) and the S1PR_4_ antagonist CYM-55380 (10 µM) as indicated. (**B**) Quantification of the autophagic marker proteins p62 and LC3-II:LC3-I in astrocytes from control (Ctrl) and SGPL1^fl/fl/Nes^ (KO) mice, cultured for 5 h in the absence or presence of rapamycin (RAPA, 5 µM) as indicated. (**C**) Representative images showing the fluorescence of the RFP–GFP–LC3 construct expressed in astrocytes in the presence of rapamycin in control (Ctrl) and KO astrocytes. For all, representative immunoblots are shown with β-actin as loading control. Bars represent means ± SEM, n ≥ 3, one-way ANOVA with Bonferroni multiple comparison test, * *p* < 0.05, ** *p* < 0.001, *** *p* < 0.0001.

Next, we set out to elucidate which of the five S1PRs is responsible for the changed expression of enzymes involved in glucose metabolism described above. As illustrated in Figure 2A, only the transcripts of S1PR_2,4_ were significantly increased in SGPL1-deficient astrocytes (Figure 2A). Note that S1PR_4_ is evidently expressed in astrocytes, although previous reports exclude this S1PR from brain tissue [36]. We want to emphasize that all of our experiments with specific S1PR_4_ agonists and antagonists undoubtedly argue for its expression and function in primary cultured cortical murine astrocytes (see Figure 2C,D, Figure 3D, and Figure 4A). In a previous study, we detected increased amounts of S1P in the culture medium of SGPL1-deficient astrocytes [29]. We now observed a twofold increase in the expression of the S1P transporter, spinster 2 (SPNS2), in astrocytes lacking SGPL1 (Figure 2B). To verify the potential effect of S1P signaling via these two receptors, we treated control astrocytes with specific agonists of S1PR_2_ and S1PR_4_ (5µM), respectively, for 24 h. As determined in our preliminary results (not shown), we found that only the combined addition of both agonists reproduced the effects of S1P shown in Figure 1D (Figure 2C).

Next, we inhibited S1PR_2_ and S1PR_4_ with JTE-013 and CYM-55380, respectively, and checked whether and how these specific antagonists affect the increased expression of the enzymes involved in glucose degradation. As shown in Figure 2D, the simultaneous addition of JTE-013 and CYM−55380 (10 µM) for 6 h reversed the expression of PFK, GAPDH, and PDH to control levels (Figure 2D). Note that, as specified above for the specific agonists, inhibition with each of the two specific inhibitors alone did not reverse the effect observed in SGPL1-deficient astrocytes, indicating that the two receptors are functionally redundant. We then determined the amount of ATP in control and SGPL1-deficient astrocytes treated with the two specific antagonists of S1PR_2_ and S1PR_4_. As shown in Figure 2E, the normalization of the glycolytic flux and of PDH expression following the inhibition of S1PR_2,4_ (Figure 2D) caused a reduction in the ATP level to control values (Figure 2E).

### 2.2. The Increased Glucose Degradation in SGPL1-Deficient Astrocytes Is Linked to mTOR Activation and Down-Regulation of Autophagy

Cellular energy metabolism and autophagy are intimately linked processes. mTOR plays a key role in the correlation of these two vital processes, acting as an integrator to support organismal and cellular interactions with the environment [37]. mTOR functions as a serine/threonine protein kinase that coordinates the availability of nutrients in conjunction with several processes that require energy, such as cellular growth, proliferation, and motility, by regulating protein synthesis, transcription, autophagy, and metabolism. Based on the increased glucose metabolization in SGPL1-deficient astrocytes, we first checked whether this metabolic change affects mTOR expression. The 4-fold increase in mTOR transcripts in these cells (Figure 3A) motivated us to examine two markers of autophagy, (i) p62, which is a specific autophagic substrate that binds to and sequesters autophagic cargo [38], and (ii) the conversion of LC3-I (microtubule-associated protein 1 light chain 3) to its lipidated conjugated form LC3-II, essential for the elongation and maturation of autophagosomal vesicles [39]. As expected, the p62 levels increased considerably (2-fold), while the LC3-II:LC3-I ratio decreased by about 40 % (Figure 3B). This result suggests dysfunctional cargo processing and the obstruction of autophagic flux. For a more accurate elucidation of autophagic flux, we transfected the RFP-GFP tandem fluorescent-tagged LC3 (RFP–GFP–LC3) construct into cultured astrocytes from control and SGPL1^fl/fl/Nes^ mice. In this tandem fluorescent-tagged autophagosomal marker, LC3 is engineered with green fluorescent protein (GFP) and red fluorescent protein (RFP), allowing the labeling of autophagosomes in yellow (by combining green and red fluorescence), while autophagolysosomes appear red after the fusion of autophagosomes with lysosomes as their acidic pH quenches GFP fluorescence. As shown in Figure 3C, a considerable number of red puncta were detected in control cells, while in SGPL1-deficient astrocytes, yellow puncta dominated (Figure 3C). This result indicates the hindrance of autophagosome/lysosome fusion caused by the elevated expression of mTOR in astrocytes lacking SGPL1 [40]. Notably, the results shown in Figure 3B could be recapitulated by the combined addition of the specific S1PR_2,4_ agonists (5 µM for 24 h) to control astrocytes (Figure 3D). This result strongly indicates that the S1P/S1PR_2,4_ axis, on the one hand, enhances glucose degradation and, on the other hand, reduces autophagic flux in SGPL1-deficient astrocytes. This finding is further confirmed by the fact that the combined treatment with the specific inhibitors of S1PR_2_ and S1PR_4_ (10 µM for 6 h) re-establish the control values of the autophagy markers p62 and LC3-II:LC3-I (Figure 4A).

Additionally, the treatment of astrocytes for 5 h with the mTOR inhibitor rapamycin (5 µM) reduced the expression of p62 and re-established the conversion of LC3-I to LC3-II in astrocytes lacking SGPL1 (Figure 4B). This result was further confirmed by the re-established autophagic flux determined by following the fate of the RFP–GFP–LC3 construct, as described above, in the presence of rapamycin (Figure 4C). Together, these outcomes demonstrate that in SGPL1-deficient astrocytes, the down-regulation of autophagy is mediated by mTOR.

## 3. Discussion

Astrocytes not only outnumber neurons by more than fivefold in the central nervous system (CNS) [41] but also critically contribute to the regulation of early neurodevelopmental processes [42]. Their essential role in normal neural activity in the healthy brain, as well as their prompt response to all forms of brain injury, is increasingly recognized [41]. In the present study, we found that S1P metabolism has a considerable impact on glucose breakdown and hence on autophagy in astrocytes. The high energy demands of the brain required to maintain the membrane ion gradients and processes related to synaptic transmission [43] are provided by ATP generated via the oxidative degradation of glucose [44]. Yet, astrocytes are predominantly glycolytic [35], extracting glucose from the blood or mobilizing glycogen stores under neuronal command [45]. S1P signaling through S1PR_2,4_ not only augmented the expression of regulatory glycolytic enzymes, including PFK and GAPDH, but also increased the expression of PDH, with a simultaneous decrease in the expression of LDH. Of interest, in non-neural cells lacking SGPL1, accumulated and released S1P also signaled (via S1PRs) an increase in glucose uptake and breakdown via glycolysis [32]. The higher expression of PFK and thus the elevation of glycolytic flux may protect SGPL1-deficient astrocytes against toxic depositions [46], which were described in SGPL1-deficient neurons [27]. On the other hand, the reduced generation of lactate due to the S1P-induced preferential degradation of glucose via the TCA cycle might negatively affect brain health [30]. Lactate generated by astrocytes has a dual role as neuronal fuel and as an intercellular messenger [45]. Thus, astrocytic lactate generated through aerobic glycolysis was shown to be essential for brain development, supporting the biosynthetic requirements of synaptic growth and remodeling [44]. Note that aerobic glycolysis, the non-oxidative metabolism of glucose despite the presence of adequate levels of oxygen, is inefficient regarding the generation of energy in the form of ATP, but beneficial in providing intermediates for the biosynthesis of lipids, nucleic acids, and amino acids [4]. In addition, lactate is considered to perform neuroprotective functions against various types of brain damage [47].

Conversely, in SGPL1-deficient fibroblasts, glycolysis was uncoupled from the TCA cycle and shifted to aerobic glycolysis, promoting cell growth similar to that often described in cancer cells [32,48,49]. In SGPL1-deficient astrocytes, however, pyruvate was preferentially channeled into the TCA cycle, thus increasing ATP formation. Like lactate, ATP also fulfills multiple functions in the brain. Thus, ATP plays an essential role as energy currency in the brain, known as the most metabolically active organ in the body [50]. Accordingly, metabolic agents that enhance ATP levels can improve cognitive functioning [50]. Yet, SGPL1^fl/fl/Nes^ mice exhibited deficits in cognitive skills, including in spatial and associative learning and memory [26,27]. It appears therefore likely that ATP is released by astrocytes, as previously described [51]. Extracellular ATP can trigger biological effects per se through the activation of P2 receptors (P2R) or through its ecto-nucleotidase-catalyzed metabolite ADP activating some P2Rs and adenosine [52]. ATP per se acts as a synaptic neuromodulator through the presynaptic regulation of neurotransmitter release and the postsynaptic regulation of other receptors or of intrinsic neuronal excitability, with an impact on synaptic plasticity [53]. As sustained high extracellular ATP levels were observed in several brain pathologies [53], it is imperative to investigate in the near future the potential effect of extracellular ATP or its metabolites in conjunction with the cognitive impairments observed in SGPL1^fl/fl/Nes^ mice.

In conclusion, S1P signaling via S1PR_2,4_, on the one hand, leads to increased ATP production but, on the other hand, reduces the formation of lactate, as indicated by the considerable reduction in LDH. All of the aspects depicted above reflect the complexity of changes induced by S1P signaling in astrocytic glucose metabolism.

**Figure 5:**
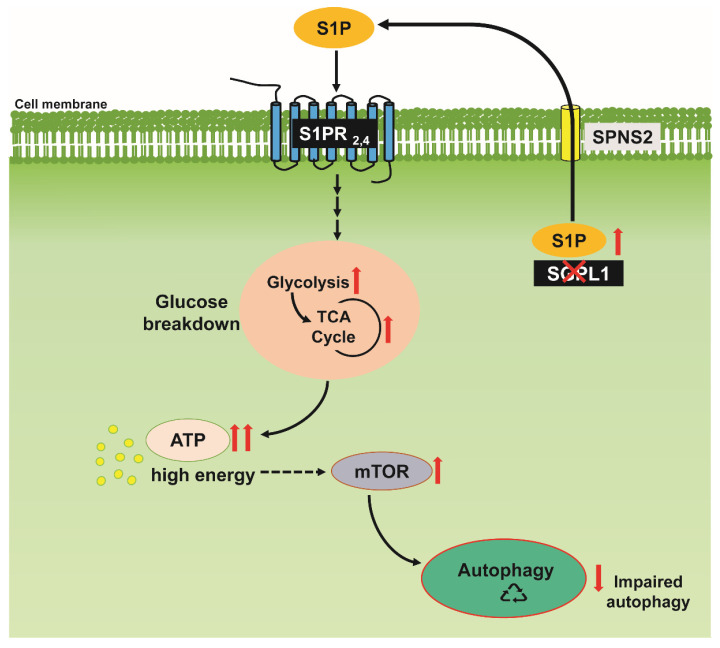
Scheme summarizing the effects of SGPL1 ablation on glucose degradation and autophagy in astrocytes. In the absence of SGPL1, accumulated S1P is released by the cells via SPNS2 and promotes the increased expression and activity of proteins involved in glucose breakdown, acting in an auto- or paracrine manner through S1PR_2,4_. This leads to increased levels of ATP, the activation of mTOR, and reduced autophagy.

Furthermore, glucose metabolism is closely associated with cellular autophagy. We indeed found that increased glucose breakdown via the TCA cycle caused the mTOR-dependent down-regulation of autophagy in SGPL1-deficient astrocytes (Figure 5). By contrast, in neurons lacking SGPL1 activity, autophagic flux was blocked due to a reduction in phosphatidylethanolamine (PE) and was mTOR-independent [27]. Importantly, we have shown before that, in SGPL1-depleted astrocytes, PE levels are not affected [29]. The crucial role of autophagy in the survival of postmitotic neurons is well established [54]. It is also well established that impaired neuronal autophagy is closely associated with almost all neurogenerative diseases [54]. A compromised autophagic process contributes to the pathogenesis of neurodegenerative diseases by hindering the clearance of intracytoplasmic deposits of aggregate-prone proteins [55]. Accordingly, we have shown that impaired neuronal autophagy in SGPL1^fl/fl/Nes^ mice causes the accumulation of aggregate-prone proteins, such as the amyloid precursor protein (APP) and α-synuclein (SNCA), associated with cognitive weaknesses in these mice [26,27]. Impaired neuronal autophagy is also a hallmark of inherited congenital “lysosomal storage” disorders causing severe neurodegenerative phenotypes [56,57]. Results similar to what was found in neurons were also reported for astrocytes affected by lysosomal storage [58]. Thus, impaired autophagosomal maturation due to lysosomal storage in astrocytes was shown to affect the survival of cortical neurons and to account for several neurological features of the disease [59]. Above and beyond this, the contribution of astrocytic autophagy to systemic metabolism has been emphasized [60]. Therefore, we cannot presently exclude a negative impact of down-regulated autophagy in astrocytes from SGPL1^fl/fl/Nes^ mice. We were able to rescue autophagy with rapamycin, which has been shown to be neuroprotective, as it overcomes all pathological effects of mTOR [61]. The consequences of SGPL1 deficiency on glucose degradation and autophagy in astrocytes (Figure 5) may contribute to a better understanding of S1P function not only in brain pathology but also with regard to the complex phenotype of patients exhibiting mutations in *SGPL1*.

## 4. Materials and Methods

### 4.1. Antibodies and Chemicals

Monoclonal antibodies against LC3 (12741), p62 (5114), PDH (3205), GAPDH (5174), PFK (8164), LDH (2012), and β-actin (4967) were purchased from Cell Signaling Technology (Danvers, MA, USA), while IDH was purchased from Sigma-Aldrich (HPA007831, St. Luis, MO, USA), and Beclin1 was purchased from Merck (SABS700251, Darmstadt, Germany). Secondary antibodies, HRP-linked anti-rabbit and anti-mouse IgG, were purchased from Cell Signaling Technology (7074 and 7076, Danvers, MA, USA). Rapamycin, CYM-5520, and CYM-50308 were from Cayman Chemical Company (13346, 17638, and 14667, respectively, Ann Arbor, MI, USA). JTE-013, CYM-55380, and S1P were procured from Sigma-Aldrich (J4080, SML1066, and SML2709, respectively, St. Luis, MO, USA).

### 4.2. Ethical Statement and Experimental Animals

All animal experiments were conducted in accordance with the guidelines of the Animal Care Committee of the University of Bonn. The experimental protocols were approved by Landesamt für Natur, Umwelt und Verbraucherschutz (LANUV) Nordrhein-Westfalen (NRW) (LANUV NRW, Az. 81–02.05.40.19.013).

The SGPL1^fl/fl/Nes^ mouse model was generated as described previously [26]. Briefly, mice harboring “floxed” exons 10–12 on both Sgpl1 alleles (SGPL1^fl/fl^) were crossbred with the nestin-Cre transgenic mouse line Nes-Cre1, in which Cre recombinase expression is under the control of the nestin promoter. We thus obtained SGPL1^fl/fl/Nes^ mice in which the “floxed” exons were excised by Cre recombinase, leading to SGPL1 ablation in neural cells. For all experiments, SGPL1^fl/fl^ mice served as controls. All mice were housed under standard conditions at the LIMES Institute of Bonn University.

### 4.3. Cell Culture

Primary astrocyte cultures were prepared as mixed glial cultures from the cortices of postnatal pups (P1 to P4). Surgical scissors were used to decapitate the pup’s neck; both skull and skin were removed following the midline with an iris scissor. The skull was removed with curved forceps, and the brain was transferred using a micro scoop into a separate Petri dish containing ice-cold HBSS buffer. In the following steps, the cerebellum and meninges were removed using forceps and transferred with the micro scoop into a 15 mL tube containing 1–2 mL of Ca^2+^- and Mg^2+^-free HBSS and kept on ice until all brains were dissected. After dissecting both control and SGPL1-deficient brains, HBSS was carefully aspirated, and 1–2 mL of 0.05% trypsin-EDTA was added. The tubes were incubated in a water bath at 37 °C for 15 min with constant shaking. To neutralize trypsin-EDTA, 1–2 mL of prewarmed cell culture medium was added, and cortices were mechanically dissociated by pipetting up and down with a sterile 10 mL pipette. Then, the tubes were centrifuged for 30 s, followed by aspirating the supernatant and adding 1–2 mL of prewarmed cell culture medium. Then, each cell suspension was loaded in T25 cell culture flasks containing 5 mL of prewarmed cell culture medium. The cells were incubated in a humidified cell culture incubator maintained overnight at 37 °C with 5% CO_2_. The next day, the culture medium was removed, and cells were washed with prewarmed sterile PBS to remove cell debris. Cells were then incubated in 5 mL of fresh culture medium, and the medium was refreshed every 2–3 days. After about 10 days, a confluent layer of astrocytes formed, with microglia and oligodendrocytes loosely growing on this astrocyte layer and being detached by vigorously shaking the flasks. Astrocytes were used for experiments after about 25 days in culture. Before astrocytes were used for experiments, microglia and oligodendrocyte precursor cells (OPCs) were detached by vigorous shaking. The medium was then removed, and astrocytes were either seeded onto 6-well cell culture dishes (35 mm diameter) or used for experiments after 24 h as needed.

### 4.4. Western Immunoblotting

The cortex was weighed, freshly prepared RIPA lysis buffer was added (three times larger volume than the tissue weight), and the samples were incubated on ice for 15 min. Following tissue softening, the tissues were homogenized using a syringe and incubated in RIPA buffer (Thermo Fisher Scientific, Waltham, MA, USA, 89900) for a further 45 min, followed by centrifugation at 14,000× rpm at 4 °C. Cell pellets were thawed on ice for 5 min, and then 150 µL of RIPA lysis buffer was added and mixed vigorously using a pipette and kept on ice for 1 h. After every 15 min, samples were vortexed for 10–20 s at maximum speed. Similar to the tissue samples, cell samples were prepared likewise and centrifuged for 45 min at 13,000 rpm, and clear supernatant (lysates) was transferred into a fresh tube. A Nanodrop (Thermo Fisher Scientific, ND-2000, Wilmington, DE, USA) was used to determine the protein concentration in the supernatants. Lysates were mixed with Laemmli buffer in a 1:4 ratio (Bio-RAD Laboratories, Munich, Germany, 1610747), and samples were heated for 5 min at 95°C before loading on SDS-PAGE gel. Proteins were separated by SDS-PAGE in running buffer (25 mM Tris, pH 8.3, 192 mM glycine, 0.1% SDS) at 50 V for 15 min and then at 150 V for 1 h. The transfer onto nitrocellulose membranes (Porablot NCL; Macherey-Nagel, Thermo Fisher Scientific, 741290, Schwerte, Germany) was performed at 4 °C and 400 mA for 2 h in transfer buffer (50 mM Tris, pH 9.2, 40 mM glycine, 20% methanol). Membranes were blocked with Blocker BSA (Thermo scientific, 37520, Rockford, IL, USA) in TBS-Tween 20 (20 mM Tris, pH 7.5, 150 mM NaCl, 0.1% Tween 20, Sigma-Aldrich, St. Louis, MO, USA, P9416) for 1 h, washed 3 times (10 min each), and incubated at 4 °C overnight with the primary antibody. Membranes were then washed three times (10 min each) and incubated for 1 h at room temperature with an HRP-conjugated secondary antibody. Western BLoT Chemiluminescence HRP Substrate (TAKARA Bio, Saint-Germain-en-Laye, France, T7101B) was used for detection with the VersaDoc 5000 imaging system (Bio-Rad, Hercules, CA, USA). β-Actin was used as the loading control. When a particular protein of similar molecular weight to β-actin was present on the blot, the blots were stripped using stripping buffer (Takara, Bio, Saint-Germain-en-Laye, France, T7135A) before being used for β-actin imaging. Quantification and statistical analysis were performed using ImageJ and GraphPad Prism programs.

### 4.5. RNA Isolation and Real-Time PCR

Up to 1 µg of total RNA (isolated with EXTRAzol from Blirt, EM30-200, Gdańsk, Poland) was used for reverse transcription with the ProtoScript^®^ II First Strand cDNA Synthesis kit (New England Biolabs, E6560L, Frankfurt am Main, Germany,). The resulting total cDNA was then applied to real-time PCR (CFX96-real time PCR, Bio-Rad) using β-actin and 18S RNA as housekeeping genes. The primers for real-time PCR were designed using the online tool from NCBI BLAST and obtained from Invitrogen. They are listed as follows (name: forward primer (for), reverse primer (rev)): β-actin, 5′-CTTTGCAGCTCCTTCGTTGC (for) and 5′-CCTTCTGACCCATTCCCACC-3′ (rev); S1PR_1_, 5′-CTACACAACGGGAGCAACAG-3′ (for) and 5′-CCCCAGGATGAGGGAGAGAT-3′ (rev); S1PR_2_, 5′-CAGGATCTACTCCTTGGTCAGG-3′ (for) and 5′-GAGATGTTCTTGCGGAAGGT-3′ (rev); S1PR_3_, 5′-CCCAACTCCGGGACATAGA-3′ (for) and 5′-ACAGCCAGTGGTTGGTTTTG-3′ (rev); S1PR_4_, 5′-TTCCATATGATGGACACTCC-3′ (for) and 5′-TGGACAAATGAACGCAGGT-3′ (rev); S1PR_5_, 5′-GCTTTCTGTGTACAGTTGACAAATACT-3′ (for) and 5′-CCAACTGTTCCAACTGTATGCT-3′ (rev); mTOR, 5′-CTGAACAGCGAGGACAA-3′ (for) and 3′-GTAGCGGATATCAGGGTCAGG-5′ (rev). The reactions were performed at 95 °C for 30 s, 95 °C for 10 s, and 60 °C for 1 min. Normalized relative mRNA expression was obtained from real-time qPCR.

### 4.6. Immunocytochemistry

After about 21 days of growth in T-25 flasks, cells were transferred on coverslips and grown for an additional 8–10 days. Following this, coverslips containing astrocytes were rinsed 3 times with PBS at room temperature and fixed in methanol (−20 °C, 5 min). The cells were always rinsed three times with PBS between each incubation step. Afterward, cells were blocked with 20% (*v*/*v*) normal goat serum in PBS for 30 min and incubated overnight at 4 °C with primary antibodies diluted to 1:200 in PBS. The cells were then incubated for 50 min at room temperature with anti-rabbit/mouse Alexa Fluor 488 (1:300)-conjugated secondary antibodies. Lastly, cells were embedded in Fluoromount G medium with DAPI for microscopic analysis.

### 4.7. Immunohistochemistry

Isolated brains were snap-frozen in liquid nitrogen. Cryo-sectioning was used to produce 10 µm sagittal sections, which were placed on Superfrost Plus positively charged microscope slides. The brain sections were fixed for five minutes in ice-cold 4% (*v*/*v*) paraformaldehyde in PBS. The sections were then permeabilized with 0.1% (*v*/*v*) Triton X-100 in PBS for 30 min at room temperature. Next, tissue sections were blocked in 20% (*v*/*v*) normal goat serum in PBS for 30 min and incubated overnight at 4 °C with primary antibodies. The primary antibodies were diluted in a 1:200 ratio in PBS containing 0.5% lambda-carrageenan (Sigma, 22049, Darmstadt, Germany) and 0.02% sodium azide and applied overnight at 4 °C to the sections. After washing, brain sections were incubated with 1:300-diluted Cy3-conjugated anti-rabbit antibody diluted in PBS with the same additions as above for 1 h at room temperature. Finally, antibody-labeled brain sections were embedded in Fluoromount G medium with DAPI for microscopic analysis (Zeiss Axioskop 2 epi-fluorescence microscope equipped with a digital Zeiss AxioCamHRc camera, Carl Zeiss Jena, Jena, Germany).

### 4.8. ATP Measurement

The concentration of ATP in the cells was determined using the Sigma-Aldrich kit (MAK190, Darmstadt, Germany). Briefly, the ATP immediately reacts with the substrate D-luciferin in the presence of luciferase to produce light. The light intensity directly represents the intracellular ATP concentration.

#### IDH Activity Test

The IDH activity test was determined using the Sigma-Aldrich kit (MAK062, Darmstadt, Germany). Briefly, in the enzyme reaction, IDH activity was measured by using isocitrate as the substrate. IDH converts NADP^+^ and NAD^+^ into NADPH and NADH, resulting in a colorimetric (450 nm) product proportional to the enzyme activity.

### 4.9. Treatment of Cells

#### 4.9.1. Rapamycin Treatment

The autophagy rescue experiments with rapamycin were carried out by treating control and KO astrocytes with 5 µM rapamycin for five hours. Rapamycin was added from a stock prepared with ethanol that had a final ethanol concentration of less than 1% in order to prevent toxicity. Control astrocyte cultures were treated with the same amount of ethanol.

#### 4.9.2. JTE-013 and CYM-55380 Treatment

Glycolysis rescue experiments were performed using JTE-013 (JTE) and CYM-55380 (CYM) treatments, which block S1PR 2 and 4, respectively. Control and KO astrocytes were incubated for 6 h with 10 µM JTE and CYM, respectively. JTE and CYM were prepared in ethanol and DMSO, respectively. The exact amounts of ethanol and DMSO were added only to control astrocyte cultures.

#### 4.9.3. S1P and S1PR_2,4_ Agonist Treatment

To confirm the role of S1P signaling, the results obtained in SGPL1-deficient astrocytes were recapitulated by extracellular administration of 10 nM S1P to control astrocytes for 24 h.

Furthermore, to elucidate the combined effect of S1PR_2_ and S1PR_4_, control astrocytes were incubated with 5 µM CYM-5520 (S1PR_2_ agonist) and 5 µM CYM-50308 (S1PR_4_ agonist) for 24 h. CYM-5520 and CYM-50308 were both dissolved in DMSO, so the exact amount of DMSO was added to the untreated astrocyte culture.

#### 4.9.4. mRFP-EGFP Tandem Fluorescent-Tagged LC3 Expression

Primary cultured astrocytes grown on coverslips were transfected with mRFP-GFP tandem fluorescent-tagged LC3 following the manufacturer’s guidelines (Thermo Fischer Scientific, Carlsbad, CA, USA, P36239). After 24 h, astrocytes were treated for 5 h with 5 µM rapamycin. Next, astrocytes were fixed with 4% PFA for 10 min. Lastly, cells were embedded in Fluoromount G medium with DAPI for microscopic analysis.

### 4.10. Statistical Analysis

The statistical analysis was performed using GraphPad Prism 9. In the figures, all values are presented as means ± SEM derived from at least 3 independent experiments unless otherwise noted. Student’s *t*-test with false discovery rate (FDR) correction or one-way ANOVA was used to estimate the significance of differences between experimental groups and controls, as appropriate. *p* < 0.05 was considered statistically significant (* *p* < 0.05, ** *p* < 0.001, *** *p* < 0.0001, **** *p* < 0.0001, compared with the respective control group).

## Data Availability

Not applicable.
